# Relations between peer bullying and adolescent depression: the mediating effect of cellphone usage

**DOI:** 10.3389/fpsyt.2025.1486628

**Published:** 2025-04-28

**Authors:** Ying Shen, Yongjie Zhou, Shuyang Ren, Yanyan Li, Yongshi Liu, Rong Zhou, Xiwang Fan, Guangping Xie

**Affiliations:** ^1^ Psychosomatic Medicine, Ganzhou Third People's Hospital, Ganzhou, China; ^2^ Shenzhen Mental Health Center, Shenzhen Kangning Hospital, Shenzhen, China; ^3^ Clinical Research Center for Mental Disorders, Shanghai Pudong New Area Mental Health Center, School of Medicine, Tongji University, Shanghai, China; ^4^ Psychiatric Clinic, Ganzhou Maternal and Child Health Hospital, Ganzhou, China

**Keywords:** peer bullying, adolescent depression, cellphone addiction, patient health questionnaire (PHQ-9), multidimensional peer victimization scale (MPVS), mobile phone addiction index (MPAI)

## Abstract

**Introduction:**

Adolescent depression is a growing concern worldwide. This study explores the relationship between peer bullying, cellphone usage, and adolescent depression, aiming to investigate a mediation effect model based on Erikson’s stages of psychological development.

**Methods:**

Using a cluster sampling method and a cross-sectional survey, a total of 2343 adolescents aged 12 to 18 were recruited from 12 national medical institutions. Cluster sampling was used to select participants who were confirmed to have depression. The survey employed the Multidimensional Peer Victimization Scale (MPVS), the Mobile Phone Addiction Index (MPAI), and the Patient Health Questionnaire (PHQ-9) for paper-and pencil evaluations. The three variables were analyzed using SPSS 26 software, applying correlation analysis and multiple linear regression analysis.

**Results:**

The results showed that peer bullying has significant positive correlation with depression (r=0.330, p<0.001) and with cellphone use (r=0.287, p<0.001). Furthermore, cellphone usage was positively correlated with depression (r=0.333, p<0.001). Additionally, cellphone usage had a partial mediating effect between peer bullying and depression (r=0.414, p<0.001, b=0.234). The results underscore the crucial role of peer relationships in adolescent psychological development. Cellphone addiction mediates the link between adolescent depression and peer bullying, highlighting its significant impact.

**Conclusion:**

These findings contribute to understanding the interplay between social influence and depression, offering practical guidelines for fostering a supportive school environment and regulating adolescents’ phone use.

## 1 Introduction

In recent years, depression has increasingly affected younger age groups, with adolescents being particularly vulnerable (1, 2). According to Chinese governmental survey by the year of 2002, the prevalence of internet use under age of 18 is 97% in China (3). According to Statistical Report on the Development of Internet in China, about 92% of adolescents under age of 18 use cellphone, 69.7% of the same population personally own a cellphone, and cellphone use is the primary device for internet access in China (4). Problematic smartphone use and or addiction is defined as a form of behavior characterized by the compulsive use of a smartphone that results in various forms of physical, psychological, or social harm (5). The problematic use of cellphones among adolescents has emerged as a significant concern (6). Multiple studies have consistently demonstrated a moderate correlation between the severity of depression and problematic cellphone use. Additionally, there is evidence linking anxiety with excessive cellphone use (7), which in turn can exacerbate both anxiety and depression in adolescents (8).

Adolescents with emotional disorders often experience cognitive and psychomotor impairments (9), such as diminished logical reasoning, attention, and memory, which can hinder or even interrupt the development of essential cognitive and learning skills (10). Depression, a serious emotional disorder and a cause of disability, manifests through physical symptoms like tiredness, weight loss, appetite loss, and psychological symptoms such as persistent sadness and loss of interest in activities (11, 12). Despite its prevalence, the etiology and pathogenesis of depression remain unclear. It is widely recognized that both environmental and genetic factors play critical roles in the development of depression (13).

Previous studies also show that emotional problems are associated with internet addiction. The underlying mechanism is that interpersonal relationships among patients with Alexitymia are challenged due to impaired recognition and response to emotions, and Alexitymia is strongly associated with depression across different population groups (14). Thus, patients with Alexithymia and depression may experience compensatory internet use, because internet provide them an ideal resort for social interaction because of its privacy, anonymity, and convenience for remote interactions (15). Social deficits and emotional disability are commonly seen among people with Alexitymia and depression. Internet becomes a platform for social and emotional support, not only because they lose the ability to understand and response to emotions, but also because their cognitive and social function is impaired. Internet addiction refers to the pathological use of the Internet by individuals, leading to adverse consequences in personal, social, and occupational life (16). Based on these reviews, the possibility of internet addiction is high among patients with depression, and it is possible that internet addiction is associated with problematic interpersonal relationships. Previous studies also prove that the relationship between bullying and internet addiction is positively associated with one another (17).

It is certainly that depression are caused by both genetic (18) and environmental factors (19). According to the “two- hit- hypothesis” (20), the cause of any psychotic disorder must have both of these two factors. One must first have a predisposed gene to mental disorders, and also experience the hit of environmental factor(s). These environmental factors include: mental stress, social nonconformity, withdrawal and other forms of diminished communication, and bullying (20). Among these factors, peer relationships are particularly influential during adolescence (21, 22). Research indicates a significant co-occurrence of peer victimization and adolescent depression (23, 24). Positive peer relationships contribute to school adjustment, academic achievement, personality development, and cognitive growth in adolescents (25), and negative peer relationships such as social isolation and peer rejection are associated with multiple mental problems such as substance abuse, juvenile delinquency, and suicidality (26). Negative peer relationships such as bullying either in friendships or romantic relationships can predict depression (27). The quality of peer relationships can influence and be influenced by adolescent mental health. Individuals with internalizing symptoms such as feelings of worry, sadness, loneliness may be more likely to withdraw from social interactions with peers, elicit negative responses from their peers, and thus become vulnerable to peer rejection, victimization and bullying (28). Peer isolation may exacerbates internalizing problems and make individuals meet the full diagnosis of depression. Based on these literature, we assume that quality of peer relations contribute to excessive internet use and the development of adolescent mood disorders. More specifically, social withdraw, peer rejection, and bullying contribute to internet addiction and psychotic problems of depression.

Bullying is defined by repetitive aggressive behavior with an imbalance of power (29). Other result of bullying on campus can lead to attention deficits, loss of learning ability, increased learning fatigue, and higher dropout rates (30). Although the influence of peer bullying on cognitive functions and depression is well-documented, there is a notable gap in research concerning its impact on depression among Chinese adolescents, especially among young internet and cellphone users.

**Figure 1:**
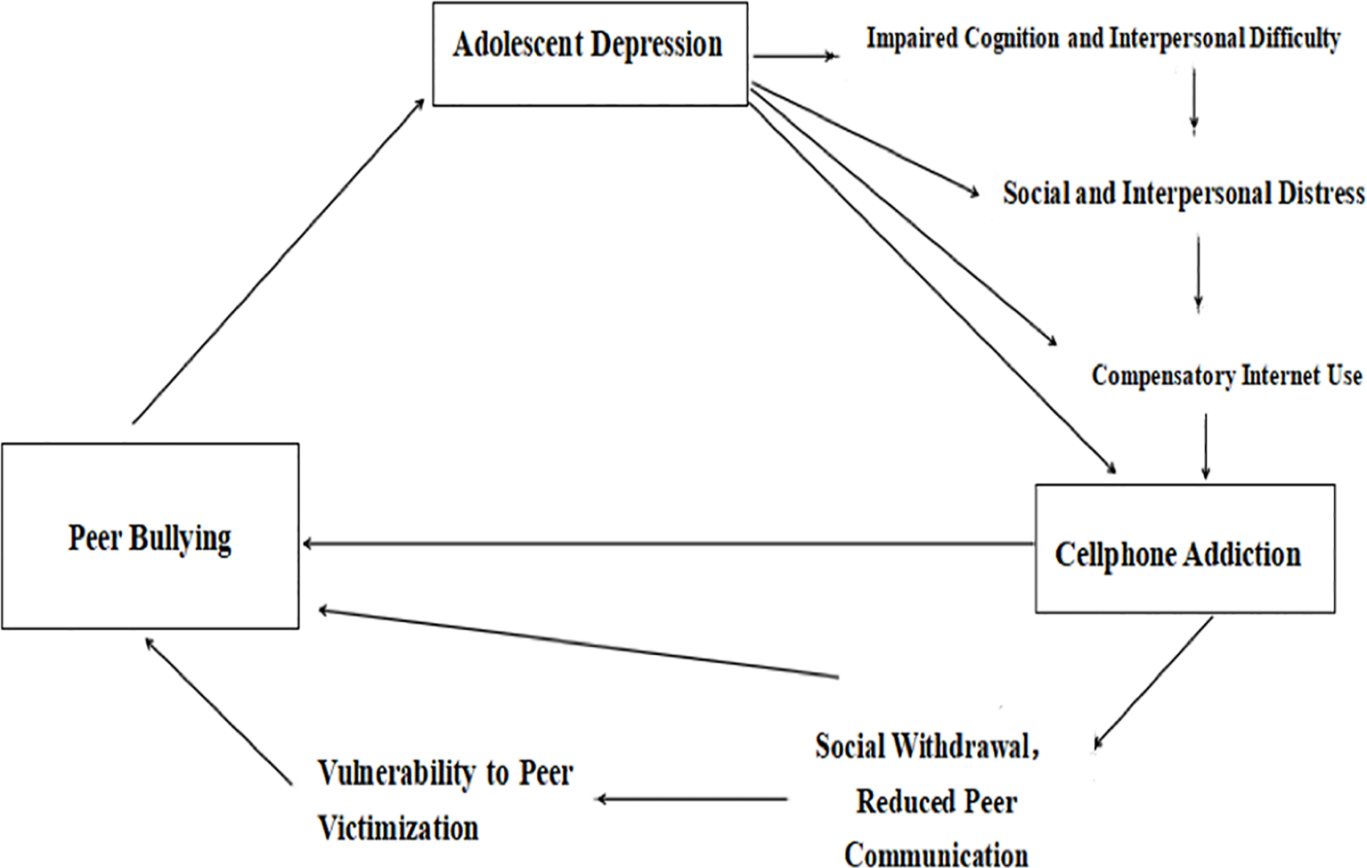
Hypothesized model diagram.

Our hypothesis is that, relationship problems, such as bullying, both verbally and physically, is not only a contributor to depression but also a contributor to cell phone addiction among Chinese adolescent population. Depression causes interpersonal difficulty and emotional distress, resulted in social withdraw, which cause impediment to integrate with peers, and vulnerability to peer victimization. Since cell phone usage are associated with both depression and interpersonal problems, it serves as a mediating factor between peer victimization and adolescent depression. **Figure 1** shows the hypothesized model diagram of our theory.

This study aims to investigate the interplay between adolescent depression, environmental factors of peer bullying, and individual factors of cellphone usage with a focus on peer bullying as the independent variable, adolescent depression as the dependent variable, and cellphone use as the mediating variable. By identifying potential moderating factors, this research seeks to offer new directions for early intervention and treatment of adolescent depression.

## 2 Method

### 2.1 Participants, sample size, inclusion and exclusion criteria

This study adopted a cross-sectional study design and conveniently sampled 2343 depression patients from 12 medical institutions across the country, ranging in age from 12 to 18 years old. Among them, there are 517 males (22%) and 1826 females (78%), with an average age of 14.99 ± 1.65 years old. The subjects were selected by the cluster sampling method. Several adolescent depression patients of different degrees based on the diagnosis of the Diagnostic and Statistical Manual of Mental Disorders, 5th edition (DSM-5). The specific inclusion criteria are as follows: (1) Adolescent patients with varying degrees of depression; (2) The age range is between 12 and 18 years old; (3) Can cooperate to complete questionnaire and other filling tasks; (4) Normal intelligence, able to use smartphones; (5) Education duration ≥ 1 year. The exclusion criteria are as follows: (1) Diagnosed as other major mental disorders except for depression; (2) Has a history of traumatic brain injury; (3) Have a history of central nervous system diseases, such as seizures, traumatic brain injury, or other organic lesions.

### 2.2 Research tools and measures

Participants were assessed on peer victimization experiences, cellphone usage, and the severity of depression using three standardized questionnaires: the Patient Health Questionnaire (PHQ-9) (31), the Multidimensional Peer Victimization Scale (MPVS) (32), and the Mobile Phone Addiction Index (MPAI) (33). The reliability and validity of these scales have been confirmed in multiple studies (34, 35).

As shown in **Table 1**, this study used these three questionnaires to evaluate whether participants had experienced peer victimization during their developmental years, their individual patterns of cellphone use, and the severity of their depression.

**Table 1:** Overview of psychomellic questionnaires used in the study.

Tool	Contents
Multidimensional Peer Victimization Scale (MPVS)	Comprising 2 1 items, this scale measures four aspects of bullying: Direct Bullying, Verbal Bullying, Social Exclusion, and Comprehensive Bullying Index. Each item is scored from 1 to 5. with a total score range of 21-105. Higher scores indicate a higher degree of bullying. The Cronbach's of MPVS in this study is 0.70.
Mobile Phone Use Questionnaire (MPAI)	Consisting of 17 questions, this questionnaire assesses the degree of cellphone addiction across four dimensions. It uses a Likert 5-point scale (1 "never" to 5 "always"), with a total score range of 17-85. Higher scores reflect a greater level of cellphone dependence. The Cronbach's a of MPVS in this study is 0.87.
Patient Health Questionnaire (PHQ-9)	Featuring 9 items, this scale evaluates the severity of depression using a Likert 4-point scale (0 “almost none” to 3 "always,"). The total score ranges from 0-27, which the higher scores indicating more severe depression. The Cronbach’s α. of MPVS in this study is 0.97.

## 3 Data analysis

Data analysis for this study was conducted using SPSS 26.0 software, following these specific steps:

First, after all participants completed the relevant scales, descriptive statistics and correlation analyses were performed on the demographic data and scale scores. This provided an overview of the sample characteristics and the relationships among key variables. We’ve collected 2464 surveys and 2343 of them are valid.

Next, linear regression analyses were carried out on the scores from the Multidimensional Peer Victimization Scale (MPVS), the Mobile Phone Addiction Index (MPAI), and the Patient Health Questionnaire (PHQ-9). These analyses aimed to evaluate the direct relationships between peer bullying, cellphone use, and depression.

To assess the mediation effect, SPSS was further used to analyze the regression coefficients sequentially, determining whether cellphone use mediates the relationship between peer bullying and depression. The validity of this mediation effect was confirmed by testing the significance of the indirect path through cellphone use.

By following these analytical steps, the study sought to elucidate the complex interrelationships among peer bullying, cellphone use, and adolescent depression.

Comparison analysis between genders for the main variables was conducted by using SPSS.

## 4 Results

### 4.1 Descriptive analysis

As shown in **Table 2**, the mean age of the 2343 patients was14.99 years (SD = 1.65). The scores on the Patient Health Questionnaire (PHQ-9) ranged from 9 to 36, with a mean score of 25.86 (SD = 7.17), indicating a wide variability in the severity of depression symptoms among the participants. The Mobile Phone Addiction Index (MPAI) scores ranged from 17 to 85, with a mean score of 45.29 (SD = 13.47), reflecting diverse levels of cellphone dependency. The Multidimensional Peer Victimization Scale (MPVS) scores ranged from 16 to 80, with a mean score of 34.55 (SD = 16.91), indicating a wide range of experiences related to peer bullying.

**Table 2:** The statistics of age and all other variables.

	N	Range of scores	Mean	*SD*
Age	2343	12-18	14.99	1.65
Patient Health Questionnaire (PHQ-9)	2343	9-36	25.86	7.17
Mobile Phone Usage Questionnaire (MPAI)	2343	17-85	45.29	13.47
Multidimensional Peer Victimization Scale (MPVS)	2343	16-80	34.55	16.91
Valid N(list wise)	2343			

### 4.2 Correlation analysis of peer bullying, adolescent depression, and cellphone use

Pearson correlation analysis, conducted using SPSS 26 software, revealed significant positive correlations among peer bullying, adolescent depression, and cellphone use. As illustrated in **Table 3**, peer bullying was positively correlated with depression (*r*=0.330, *p*<0.001) and cellphone use (*r*=0.287, *p*<0.001). Additionally, cellphone use was positively correlated with depression (*r*=0.333, *p*<0.001).

**Table 3:** Correlation analysis of peer bullying, adolescent depression, and cellphone use.

	Peer Bullying	Depression	cellphone Use
Peer Bullying	1	.330^**^	.287^**^
Depression		1	.333^**^
cellphone Use			1

*n*=2343, **p<.001

### 4.3 Regression analysis of peer bullying, adolescent depression, and cellphone use

**Figure 2:**
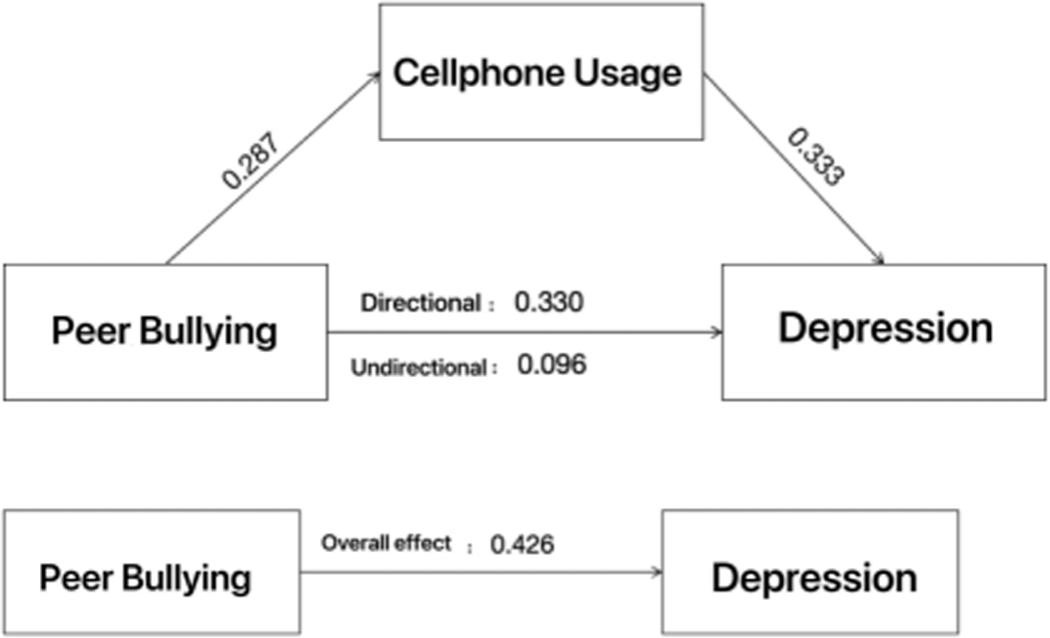
Strong Associations between peer bullying, cellphone use, and depression.

A regression analysis conducted using SPSS 26 software revealed that peer bullying had a significant positive impact on both cellphone use and depression. As shown in **Figure 2**, the direct effect of peer bullying on depression was 0.330, the indirect effect was 0.096, and the total effect was 0.426, illustrating the strong associations between these variables.

### 4.4 The mediating effect of cellphone use on peer bullying and adolescent depression

Using SPSS 26 software and Sobel test, it was found that cellphone use significantly mediates the relationship between peer bullying and adolescent depression (*r*=0.414, *p*<0.001). This suggests that peer bullying partially influences adolescent depression through increased cellphone use, highlighting the importance of addressing cellphone dependency in efforts to mitigate the effects of bullying on adolescent mental health.

### 4.5 The comparison of gender difference

**Table 4** shows the results of the t- test of the gender difference between three variables. As it shows, the associations between adolescent depression, cellphone usage and peer victimization don’t have a significant gender difference (p>0.05).

**Table 4:** Descriptive Statistics and Gender Differences of Key Variables.

	Total Sample (*n*=2343)		Male (*n*=517)		Female (*n*=1826)		t	|Cohen’s d|
	*M*	*SD*	*M*	*SD*	*M*	*SD*		
MPVS	34.55	16.91	33.3	17.07	34.91	16.85	0.78*	0.095
PHQ9	25.86	7.17	15.22	7.28	17.32	7.07	0.10*	0.295
MPAI	45.29	13.47	44.99	13.12	45.37	13.57	0.19*	0.028

MPVS, Multidimensional Peer Victimization Scale; PHQ9, the Patient Health Questionnaire; MPAI, Mobile Phone Use Questionnaire; *P>0.05

## 5 Discussion

This study identified a significant positive correlation between peer bullying and adolescent depression, with cellphone use partially mediating this relationship. These findings align with existing research, which underscores the substantial negative impact of peer bullying on adolescent mental health, particularly when compounded by cellphone addiction. Our finding confirmed our hypothesis that depression caused impaired cognition among adolescents, and further resulted in peer relational problems, social and interpersonal difficulties, social withdrawal, internet addiction, vulnerability to peer victimization, peer bullying, and ended up exacerbating the symptoms of depression. It also provided more information for previous literature about the relationship between negative peer relation and depression. Our study confirms that while internet usage serves a mediating factor, negative peer interaction, peer victimization and bullying directly exacerbate internalizing problems, and as a result cause adolescent depression. The underlying logic is that, the more severe the depression is, the longer the time adolescent spend time on internet and cellphone because of impaired social, cognitive and interpersonal function. The more time adolescent spend online and on cellphones, the less time they spend with their peers. As a result, they would experience peer rejection and social isolation, and cause interpersonal misunderstanding. Interpersonal misunderstandings cause tensions and jeopardize any kind of peer relationships, and eventually engender the happening of peer victimization. Moreover, because of depressive symptoms, the ability of interpersonal communication and self-explanation is also damaged, rendering internet addicts socially helpless. Victims are unable to rebuild and reform any new peer relationships, so longer social isolation and chronic peer victimization happen. Therefore, compensatory internet usage is not only a result of depression, but also a dangerous trigger to depression among adolescents. Compensatory internet use may develop into an addictive behavior, consume more social interaction time, and further resulted in peer isolation, peer rejection, victimization and finally increase the possibility of bullying victimization, severe internalizing symptoms, feelings of anxiety, loneliness, sadness, etc. These aftereffects of internet use eventually exacerbates depressive symptoms and met the full criteria of depression in the DSM-5.

Adolescence is a critical stage in an individuals socialization process, where peer relationships significantly influence psychological development (36, 37). This study confirms that negative peer interactions, such as bullying, can lead to social withdrawal, escapism into the virtual world, and the subsequent development of cellphone addiction and depression (38, 39). These issues interfere with adolescents role identity formation, leading to role confusion and adversely affecting their personality and mental development (40, 41).

Moreover, the overuse of cellphones may serve as a mediating factor between anxiety and depression. Adolescents who excessively use their phones are at risk of becoming addicted to social media and online games (42), which may cause them to neglect real-life social interactions. This excessive dependence on cellphones can lead to feelings of anxiety and loneliness, further increasing the risk of peer victimization, bully and depression.

Even though 78% of samples in this study are female students, our statistic result shows that our theory does not have a gender bias. Internet usage does same harm to male adolescents as it does to female adolescents in terms of triggering depression and further cause peer victimization.

A global survey conducted in 2022 revealed that from 1990 to 2019, the global disability-adjusted life year ratios attributable to anxiety disorder and major depressive disorder among victims of bullying increased by 23.31% and 26.60%, respectively (43, 44). These statistics further emphasize the critical role bullying plays in the development of mental disorders among victims.

Since peer victimization can both predict adolescent depression and cellphone addiction, prevention to peer victimization can both prevent the formation of depression and cellphone addiction. Schools and educators can utilize a web-enabled, school-based intervention for bullying prevention (LINKlusive). This intervention is proved to show significant effect on depression among students with peer-reported bullying victimization (45). Other anti-bullying programs such as KiVa in United Kingdom is also proved to be useful in reducing bullying victimization (46). Based on the finding of this study, we assume that prevention likewise will also reduce cellphone usage and mitigate the harm cellphone usage does to student’s learning and cognitive ability.

### 5.1 Limitations

This study has several limitations, including the use of a convenience sample and a cross-sectional design, which limit the ability to establish causal relationships. Future research should consider adopting a longitudinal design to better explore the causal relationships between peer bullying, cellphone use, and depression. Longitudinal studies are needed because studies have found that victims of frequent bullying suffers from more severe adverse outcomes than victims of occasional bullying (47). As a result, victims with chronic bullying may develop more serious internet addiction and suffer from more severe depressive symptoms than victims with occasional bullying. Additionally, while this study examines the mediating role of cellphone overuse between bullying and depression, it does not account for the potential influence of internet bullying encountered through cellphone use, which could serve as a confounding variable. Cyber bulling such as gossiping, rumor spreading, group, personal and sexual harassment can also be other ways of peer victimization, because they all meet the definition of bullying. In the context of school environment, accessibility to internet is convenient once students own his or her cellphone, so cyberbullying could be another form of bulling and another alternative mediator. In this study, the Multidimensional Peer Victimization Scale (MPVS) doesn’t have a index of cyber bullying, so the effect of internet bullying cannot be measured. Further studies need to utilize another tool to measure the influence of cyber bullying. Previous studies have discussed about confounding variables contribute to peer victimization. Previous researches have confirmed that variables such as social economic status can contribute to peer victimization among Indian population (48). Other confounding variables may be family attachment, parenting styles, self-esteem (49), access to digital devices, psychological resilience and family mental health history. Controlling for these confounding variables in future studies may reflect a more salient association between each two of these three variables in this study. While thinking about controlling these variables, measurement is necessary. Some of the measurement tools are Inventory of Parent and Peer Attachment for Children and the Rosenberg Self-Esteem Scale (49), and Connor-Davidson Resilience Scale, (CD- RISC) (50). Another limitation of this study is the statistical method, future studies could think about using more complex statistical method such as structural equation modeling or path analysis to capture the complicity of the relationships between the variables in this study. Finally, the population group is limited to Asian population, especially Chinese young adolescents, so it doesn’t cover a wider group population. Later studies can focus on different population groups such as the European, North and South American, Hispanic, and African population.

### 5.2 Conclusion

Peer relations play a critical role in youth psychopathology. The findings indicate that negative relationships, such as bullying in all its forms, significantly contribute to adolescent psychosis, particularly depression. These results underscore the need for further studies to explore the relationship between bullying victims and other psychotic disorders, such as anxiety, sleep disorders, and personality disorders. Additionally, cyber-bullying should be taken into consideration, as increased cellphone use may elevate the risk of encountering verbal attacks online, potentially contributing to depression. Future research should also aim to determine which types of bullying behaviors have a stronger correlation with depression. Understanding these dynamics will be crucial for developing targeted interventions and preventive measures. In conclusion, this study highlights the profound impact of peer relations on adolescent mental health and emphasizes the importance of addressing various forms of bullying to mitigate their adverse effects. Furthermore, it sets the foundation for studies of multi-relational model between addictions, mood disorders and behavioral problems among other adolescent groups.

## 6 Future directions

Further studies need to explore the relationship between cyber bullying and depression, cyber bullying and internet addiction, for cyber bullying could be another mediating effect on the relationship between adolescent depression and cellphone usage. Longitudinal studies about relationship between chronic peer victimization and adolescent depression is another concern for future researches. Further studies could provide more information about internet usage and cellphone usage behavior, and their implication to depression within another population group. For intervention purposes, future studies can consider the role physical activities play on the intervention of peer bullying and internet addiction. Previous studies have shown that physical activities can reduce the possibility of bullying victimization (51). Physical activities is also beneficial to maintain cognitive function for postmenopausal women (52). Exploration on the intervention effects of physical activities on adolescent depression and internet addiction is important. Perhaps physical activities can have a moderating effect on the relationship between depression and peer victimization. Moreover, the average time each participant spend on his or her cellphone in this study is 3.2 hours a day. For students between age of 12-18, the time spend on cellphone must be lower than 3 hours per day in order to prevent depression and peer victimization. Further studies also need to explore the appropriate time adolescent spend on cellphone each day.

## Data Availability

The raw data supporting the conclusions of this article will be made available by the authors, without undue reservation.
